# Assessment of Rabies Exposure Risk in a Group of U.S. Air Force Basic Trainees — Texas, January 2014

**Published:** 2014-08-29

**Authors:** Bryant J. Webber, Karyn J. Ayers, Brad S. Winterton, Heather C. Yun, Thomas L. Cropper, Johnnie Foster, Matthew C. Kren, Brianna Y. Meek, Tiffany A. Oliver, Christopher M. Hudson

**Affiliations:** 1 Trainee Health Surveillance, 559 MDOS, Joint Base San Antonio (JBSA)-Lackland; 2 Aerospace Medicine Department, 559 AMDS, JBSA-Lackland; 3 Public Health Department, 559 AMDS, JBSA-Lackland; 4 San Antonio Military Medical Center, JBSA-Fort Sam Houston.

In January 2014, members of the Joint Base San Antonio (JBSA)-Lackland, Texas, preventive medicine and public health teams evaluated a U.S. Air Force basic training squadron for potential exposure in sleeping bays to rabies virus carried by Mexican free-tailed bats (*Tadarida brasiliensis*). Exposure to bats while asleep or otherwise unaware is an important risk factor for rabies in the United States. Over the past several decades, most indigenous human rabies infections in the United States have resulted from the bite of an infected bat (1), and the bite was not reported in more than half of the cases (2). Mexican free-tailed bats in Texas often carry rabies virus. Among 8,904 bats tested during 2001–2010, a total of 1,558 (18%) tested positive for rabies (3). To assess the risk to the Air Force trainees and identify those for whom rabies postexposure prophylaxis (PEP) might be indicated, Lackland preventive medicine and public health teams interviewed 922 persons (866 trainees and 56 instructors) and determined that PEP, consisting of human rabies immune globulin and the 4-dose vaccination series given over 14 days (1,4), was indicated for 200 persons (22%). This report describes the public health response to a mass indoor exposure to bats, including group-based rabies risk stratification, adverse reactions to PEP, and infestation remediation. These interventions can be considered for future mass exposures to bats.

## Investigation and Results

Each year, approximately 35,000 recruits join the U.S. Air Force by completing the 8.5-week basic military training course at JBSA-Lackland in San Antonio, Texas. Because of aging infrastructure, new buildings are being constructed on the base for basic training. However, currently some of the training squadrons inhabit new buildings and some are housed in older buildings. Each of the older buildings has 20 dormitories, and each dormitory accommodates one flight of up to 60 trainees in two large sleeping bays (Figure); a flight is the smallest organizational unit in the U.S. Air Force.

On January 17, 2014, during an inspection of an older building for an unrelated health concern by the Lackland preventive medicine and public health teams, a few trainees mentioned seeing bats in their sleeping bays on multiple nights while trainees were asleep. Based on recommendations from the Advisory Committee on Immunization Practices (1) and with the consensus of military and civilian experts, it was determined that the 45 trainees currently living in the affected dormitory would require rabies PEP. Trainees in this “index flight” received their first dose of vaccine that evening and received human rabies immune globulin 3 days later, after sufficient local supply was established.

Later on January 17, the investigative team determined that bats might have been seen in six additional dormitories within the same building. Applying ACIP recommendations (1) and drawing on the experience of mass bat exposure investigations by the Kentucky Department for Public Health (5) and the Kansas Department of Health and Environment (6), the team interviewed 866 trainees to assess their exposure risk. Because trainees sleep in open bays, and thus would have similar exposures, flight-based risk category criteria were established in conjunction with CDC and the state health department (Table). Rabies PEP was recommended for all trainees in flights meeting criteria for increased risk (moderate risk or high risk).

Interviews determined that bats had been observed in seven of the building’s 20 dormitories at various times during December 22–January 16. The majority of exposures were reported by trainees in the index flight in the week preceding the investigation (January 10–16), and this flight was considered at high risk. Three additional flights were found to be at moderate risk, and the remaining 14 flights were determined to be at low risk; two other dormitories were not occupied.

Individualized risk assessments were performed on trainees who had previously moved out of the building. Of 35 trainees who might have been exposed but were not currently residing in the building (19 had been moved to medical hold, 15 had separated from training, and one had been hospitalized for an unrelated condition), it was determined that nine were at increased risk and required PEP. Three of these nine trainees reported direct contact with a bat, but none reported a bite.

Rabies PEP was initiated on January 20 for 157 trainees not in the index flight who were found to be at increased risk for exposure. For two trainees, the series was discontinued after the first dose when the two were reclassified as at low risk in accordance with the criteria established (another trainee, with documented previous receipt of rabies preexposure prophylaxis, received 2 vaccine doses and no human rabies immune globulin). In all, 200 trainees (45 in the index flight plus 155 of 157 not in the index flight) completed the rabies PEP series during January 20–February 3. PEP was not indicated for any of the instructors.

Adverse reactions included urticaria (two cases) and pruritus (six), which approximate the respective incidence rates of 1.6% and 3.3% previously reported (7). Symptoms began within 15 minutes of vaccine administration for all persons; there were no reactions to human rabies immune globulin alone. Of those with urticarial reactions, one had rapid resolution with oral diphenhydramine, and the other required intramuscular methylprednisolone and intramuscular diphenhydramine. The latter person experienced a more intense reaction after the second vaccination and was treated with an oral steroid taper and H2 blocker to complete the series. The remaining six persons had mild reactions that resolved with oral diphenhydramine.

Although the dates of the bat sightings were well confirmed, the team extended the investigation retrospectively 1 year given the prolonged incubation period for rabies (1). After interviewing 56 training instructors in the squadron and reviewing all pest control requests made to civil engineering in the past year, no previous exposures were identified. One Mexican free-tailed bat captured during the investigation tested negative for rabies.

The investigative team facilitated ongoing and direct communication with the trainees, training leaders, and local media to describe rabies transmission risk, rationale for PEP, and expectations for injections. Trainees were allowed to telephone a family member to describe the situation.

## Bat Infestation and Remediation

The installation’s civil engineering unit was notified of the bat sightings early in the investigation. Working with a commercial bat control specialist, they determined, based on guano, urine, and grease markings, that a small colony of bats (estimated at 400–600) had been nesting inside wall crevices of the building for several years. Although it is unclear why they only recently entered the dormitories, it was speculated that an unseasonably warm period halted the typical southward migration of bats from southern Texas. The return of cold temperatures caused the bats to seek warmth, and they entered the dormitories through porous structures.

Building remediation took several days, during which time trainees in the affected dormitories were relocated. The commercial bat control specialist completed the risk assessment, and civil engineers sealed gaps where bats could gain entrance to the sleeping quarters. Air vents were covered with a fine wire mesh, fastened with sheet metal, and sealed with metal tape. Damaged ceiling tiles were replaced and fastened with metal tape. Fissures in walls or between floor materials were caulked or covered with foam and sealed with metal tape. Windows with inefficient locking mechanisms were secured. One-way exclusion devices were installed to allow any remaining bats to leave but not return. A postremediation inspection verified proper sealing of entry points, and after 2 weeks of daily inspections for bat sightings, the exclusion devices were removed and the building was declared clear for reoccupation. The other occupied older buildings were inspected and considered free of bats.

### Discussion

This is the largest military investigation of rabies exposure, both in terms of the number of persons interviewed for potential exposure (922) and the number who received rabies PEP (200), with an associated cost of approximately $400,000. Careful adherence to current building codes likely is more than adequate to prevent bat infestations in new buildings. For older structures, which might have been built under less stringent codes and have become porous over time, routine inspections by animal control specialists for bat and other animal infestations are recommended.

Rabies is a zoonotic disease that causes an acute encephalomyelitis and is almost always fatal if PEP is not administered before symptom onset. PEP is recommended for persons who are bitten or scratched by a bat and that bat is either unavailable for testing or tests positive for rabies. It is also recommended for persons who might have had an exposure they are unaware of (e.g., waking in a room with a bat or being near a bat while having a condition that might result in a lack of awareness of a bat bite) (1). Military trainees, who have multiple reasons for altered sleep, might be at increased risk for an undetected bat bite while sleeping.

The discovery of bats in the training building was made by health personnel responding to an unrelated concern. Despite numerous bat sightings involving multiple trainees and instructors over several days, no one had reported them to public health authorities. This supports previous reports that document an underappreciation of the health risks associated with indoor bat exposures (5). The degree of risk was conveyed at subsequent meetings with military leaders and to all incoming trainees. No known rabies infections have ever been acquired during U.S. Air Force basic training. The death of a recent Lackland graduate in 2011 was attributed to raccoon-variant rabies not found in Texas; the trainee likely was infected while animal trapping in North Carolina before enlisting in the military (8).

This investigation benefited from early and consistent collaboration with logistics, civil engineering, and training leadership staff members, and from consultation with various military and civilian experts. Risk assessments of previous mass bat exposures (5,6) were used to guide the response. Because at least four such mass exposure episodes have been reported since 2010 (5,6,9), it might be beneficial to have a formal, national guideline outlining the appropriate strategy in these situations. Risk assessments and decisions regarding rabies PEP should be conducted in consultation with appropriate public health or preventive medicine authorities.

What is already known on this topic?

Human rabies infections in the United States, although rare, typically result from contact with a bat. Exposures to bats while sleeping comprise a large percentage of these cases. Occasionally these exposures happen on a massive scale, such as when bats are found in large, shared sleeping quarters.

What is added by this report?

A total of 922 U.S. Air Force basic trainees and training instructors were interviewed for potential exposure to bats, and 200 trainees received rabies postexposure prophylaxis (PEP). Given the shared exposure status of trainees sleeping in common bays during basic training, this investigation relied on group-based, rather than individual-based, risk assessments, using a questionnaire developed in collaboration with CDC and state authorities.

What are the implications for public health practice?

Older buildings might be susceptible to bat infestations. Periodic environmental assessment and bat remediation can prevent exposure of persons to bats and decrease the need for rabies PEP. Exposure risk assessment and decisions regarding PEP should be conducted in consultation with appropriate public health or preventive medicine authorities.

## Figures and Tables

**FIGURE:**
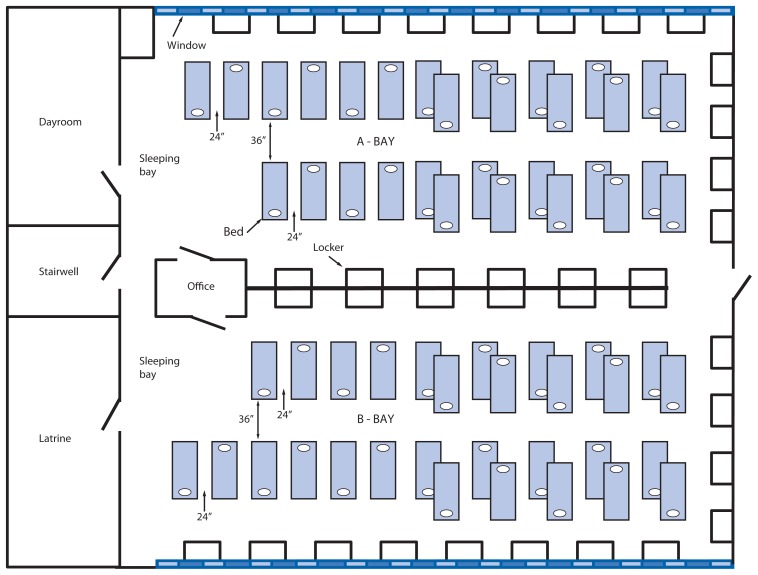
Floor plan of a dormitory housing one flight* of U.S. Air Force basic trainees — Joint Base San Antonio (JBSA)-Lackland, Texas, January 2014 * A flight is the smallest organizational unit in the U.S. Air Force. At Lackland there were 50 trainees in each flight, occupying two sleeping bays of each dormitory.

**TABLE:** Flight-level* risk category criteria used in an assessment of rabies exposure risk among U.S. Air Force basic trainees — Joint Base San Antonio (JBSA)-Lackland, Texas, January 2014

Risk category	Category criteria
High	Vast majority (>75%) of the flight woke up to find one or more bats in the sleeping bay *and* this occurred on ≥2 nights *and* interviews were totally corroborative.
Moderate	A proportion (1%–75%) of the flight woke up to find one or more bats in the sleeping bay *and* this occurred on ≥1 night *and* interviews were mostly corroborative.
Low	No one in the flight woke up to find a bat in the sleeping bay *and* interviews were totally corroborative; *or* a proportion of the flight originally reported waking up to find a bat in the sleeping bay, but upon further questioning it was concluded that the bat was observed for the entire time it was in the bay.

* A flight is the smallest organizational unit in the U.S. Air Force. At Lackland there were 50 trainees in each flight, occupying two sleeping bays of a dormitory.
